# Validation of an Automated Cell Counter Method for HLA-DR and CD3 Expression in Cells Obtained from Low Volume Human Tears

**DOI:** 10.3390/diagnostics15091124

**Published:** 2025-04-28

**Authors:** Carmen Ciavarella, Annalisa Astolfi, Chiara Coslovi, Michele Potenza, Gianandrea Pasquinelli, Luigi Fontana, Piera Versura

**Affiliations:** 1 DIMEC, Department of Surgical and Medical Sciences, Alma Mater Studiorum University of Bologna, 40138 Bologna, Italy; carmen.ciavarella2@unibo.it (C.C.); annalisa.astolfi@unibo.it (A.A.); michele.potenza2@studio.unibo.it (M.P.); gianandr.pasquinelli@unibo.it (G.P.); 2 IRCCS Azienda Ospedaliero-Universitaria di Bologna, 40138 Bologna, Italy; chiara.coslovi@unibo.it (C.C.); luigi.fontana6@unibo.it (L.F.); 3 Ophthalmology Unit, DIMEC, Alma Mater Studiorum University of Bologna, 40138 Bologna, Italy

**Keywords:** tears, inflammation, microquantities volume, automated cell counter

## Abstract

**Background/Objectives**: Tears are a promising source of biomarkers reflecting both ocular and systemic conditions. However, small sample volumes and low cell yields pose technical challenges in analytical workflows. This study aimed to evaluate the feasibility of quantifying total cell counts and characterizing HLA-DR and CD3 expression in tear-derived cells using an automated cell counter with fluorescence detection (Countess 3 FL). **Methods**: Tears were collected from 31 patients, centrifuged and the resulting pellet was incubated with HLA-DR and CD3 antibodies, markers of inflammation and T lymphocytes, respectively. Data obtained from Countess 3 FL were compared with conventional flow cytometry and immunofluorescence. For technical performance analysis, precision and reproducibility of cell count and staining were measured. For method validation, an in vitro model of hyperosmolar stress was assessed by culturing conjunctival epithelial cells (CCL20.2) with 350 or 450 mOsm NaCl. **Results**: The total cell yield in each tear sample correlated with the tear surnatant volume, in a range of 1–40μL (mean total cell number: 1.3 ± 1.1 × 10^4^, correlation analysis with tear volume: r = 0.47, *p* < 0.05). HLA-DR and CD3 were detected in all samples, with a mean value, respectively, of 43.6% (±21.0) and 25.0% (±15.0) intensity. Data were comparable to those obtained from standard flow cytometry analysis.HLA-DR increase in CCL20.2 exposed to hyperosmolar stress was recorded using Countess 3FL reading, confirming the detection capacity of the proposed method. **Conclusions**: The automated cell counter can provide HLA-DR and CD3 quantification in tear cell samples, despite the high variability and the low volume availability of tear samples. Method standardization and technical improvements are necessary to strengthen this application in the clinical setting.

## 1. Introduction

Ongoing research is increasingly focused on expanding the repertoire of biomarkers and analytical technologies for improved disease detection, staging and personalized therapeutic approaches. In this context, alternative and more accessible biological fluids, beyond conventional blood samples, are under investigation, with tears emerging as a particularly promising candidate [1]. The clinical relevance of tear composition is gaining attention, as mounting evidence highlights its close correlation with the physiological and pathological status of the ocular surface [2]. Tears play essential roles in maintaining ocular surface homeostasis, providing lubrication, removing debris, delivering nutrients and serving as a first-line defense barrier [3]. Alterations in tear quantity, composition, or stability are directly implicated in the pathogenesis of ocular surface diseases, most notably dry eye disease (DED) [4].

However, the limited volume and low cellular content of tear samples may compromise the quality, reliability and diagnostic utility of the analyses, thus highlighting the need for more sensitive, user-friendly and automated analytical platforms.

In the present study, we attempted to characterize the inflammatory profile of tear cell pellets. Inflammation is recognized as a key factor in the pathogenesis of dry eye disease [5,6], as it contributes to tear film instability and ocular surface damage, sustaining a self-perpetuating inflammatory cycle. The evaluation of inflammatory biomarkers in tear samples, represents a valuable tool for disease stratification, early diagnosis and therapeutic monitoring [7,8]. Among others, the expression of the human leucocyte antigen-DR (HLA-DR) [9] and the involvement of different T lymphocytes subsets have been proposed [10].

The analysis was performed through an automated image-based cell counter (Countess 3 FL, Thermo Fisher Scientific Inc., Segrate, Italy). This is a benchtop instrument addressed at automatic cell counting, that can be equipped with EVOS light cubes for fluorescence detection. Flow cytometry represents the gold standard for immunophenotyping, but the microquantities of tear sample might affect the reliability of data analysis and interpretation. Ease of utilization, immediacy in data interpretation and capacity to read small sample volumes are objective advantages of an automated cell counter that might bridge the gap between the growing diagnostic role of tear-based analysis and their technical limitations for a shift in the clinical practice.

## 2. Materials and Methods

### 2.1. Study Population and Study Design

Tears are routinely collected at our center and processed for protein electrophoresis to detect major tear protein content [11,12]. The present study was performed on pellets of cells obtained after centrifugation of tears otherwise routinely wasted from 31 patients (12 males, 19 females mean age 58.2 ± 8). All samples were treated anonymously and the protocols used in this study were implemented in accordance with the Helsinki Declaration and were reviewed and approved on 16 May 2024 by the ComitatoEtico Area Vasta Emilia Centro dellaRegione Emilia-Romagna (CE-AVEC)—protocol code 255/2024/Oss/AOUBo.

### 2.2. Tear Collection and Analysis by Countess FL Automated Cell Counter

Tears were collected by micropipette aspiration from the lower fornix in 0.5 mL tubes and immediately processed. After measuring the initial tear volume, samples were centrifuged at 13,000 rpm for 15 min and the resulting pellet was processed for cell count and characterization by Countess 3 FL, immunofluorescence and flow cytometry.

For flow cytometry and Countess 3 FL analysis, tear pellets were resuspended in bovine serum albumin (BSA) at 1% in phosphate saline buffer (PBS) in a final volume of 100 μL and incubated with the following conjugated antibodies: PE anti-human leukocyte antigen-DR as marker of inflammation (1:50, clone L243, BioLegend, San Diego, CA, USA) and FITC anti-human CD3 as marker of T cells (1:100, clone HIT3a, BioLegend, San Diego, CA, USA), according to the manufacturer’s instructions.After 1 h incubation at dark at 37 °C, cell yield and antigen positivitywasanalysed by theCountess 3 FL Automated Cell Counter (Thermo Fisher Scientific Inc., Segrate, Italy), following the manufacturer’s instructions. The protocol used for the analysis took account for the following measurements: brightfield for cell count, green fluorescence for CD3 expression and red fluorescence for HLA-DR expression. A gating based on cell size range between 9 and 30 μm was established for each analysis, takingthe conjunctival cell dimensions into account [13].

For measuring the method performance, intra-assay (repeatability) and inter-assay (reproducibility) precision were evaluated. For repeatability, two different measurements of three different tear samples were performed. For reproducibility, two different measurements of three different tear samples were performed by two independent researchers. Coefficient of variation (CV) was obtained by dividing the standard deviation (SD) on each mean value of measurements.

### 2.3. Immunofluorescence

Before expression analysis of HLA-DR and CD3 by Countess 3 FL, the antibody detection in tear cell pellets was performed by double immunofluorescence. To this aim, 20 μL of tear cell pellet were smeared on glass slides, fixed with Ethanol 100% and stained with HLA-DR and CD3 antibodies, for 1 h at room temperature, at dark. Then, slides were washed with PBS and nuclei were counterstained with 4′,6-diamidino-2-phenylindole, dihydrochloride (DAPI, Thermo Fisher Scientific).

### 2.4. Flow Cytometry

The characterization of tear pellets for HLA-DR and CD3 expression was further performed by flow cytometry to verify and validate results obtained by Countess 3 FL. To this aim, the Image Enabled Acoustic Fosusing Cytometer Attune Cytpix (Thermo Fisher Scientific Inc., Segrate, Italy) was used and kindly provided by Life Technologies Italia—Segreen Business Park (Segrate, Italy). Comparison with flow cytometry data were also obtained with joint analyses on the same samples evaluated with the BD FACSCanto II (BD Biosciences, San Jose, CA, USA).

### 2.5. In Vitro Model of Ocular Surface Damage

An in vitro model of dry eye disease was assessed by exposing conjunctival epithelial cells (clone 1-5c-4 [Wong-Kilbourne derivative (D) of Chang conjunctiva] cell line, ATCC) at increasing concentrations of sodium chloride (NaCl) for hyperosmolar stress induction. To this goal, 5 × 10^4^ cells were seeded in 24-well plates and after 24 h were treated with 350 milli osmolar (mOsm) NaCl to simulate a mild dry eye and 450 mOsm NaCl for severe dry eye condition. After 24 h, cells were collected and incubated with HLA-DR and CD3 antibodies in BSA 1% buffer for 1 h at 37 °C, as described above. Cell counts and GFP/RFP positivity were measured by the Countess 3 FL.

### 2.6. Statistical Analysis

Cell counting was determined on two different fields for each tear sample and reported as the mean of the two different measures. Data analysis, graph elaboration and statistics were performed by using Graph Pad Prism software version 10 (GraphPad Software, LaJolla, CA, USA), and MedCalc version 22.016 (MedCalc software Ltd., Ostend, Belgium). Data are presented as mean ± standard deviation. Correlation, regression and Bland–Altman analyses were conducted. A *p*-value of less than 0.05 was considered statistically significant.

## 3. Results

### 3.1. Cell Yield of Human Tear Samples by Countess 3 FL

**Figure 1:**
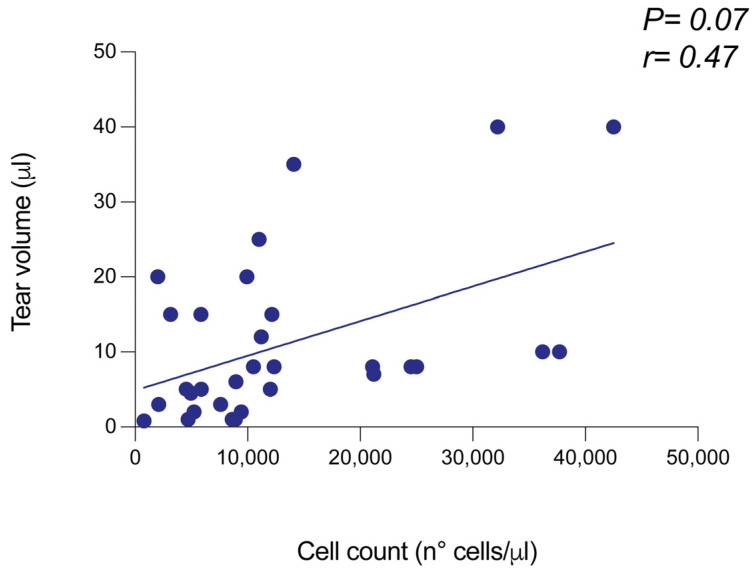
Performance of Countess 3 FL at measuring the total cell yield from pellets derived from human tear samples. The cell yield of each pellet obtained by tear sample centrifugation was analyzed by automated cell counter Countess 3 FL, by using the brightfield filter. The cell amount was provided in terms of cell number/mL and then related to the volume of pellet dilution (100 μL). A correlation test was performed between the tear volume before centrifugation and the total cell number.

The human tear cell yieldwasmeasuredon pellets obtained from 31 samplesby Countess 3 FL. According to the brightfield analysis, a mean value of 1.3 × 10^4^ cells (±1.1 × 10^4^) was obtained (minimum value: 0.78 × 10^3^ cells; maximum value: 4.2 × 10^4^ cells). The wide range of cell measurement values depended on the high biological variability existing among patients and the broad spectrum of clinical settings. Considering the impact of disease conditions on tear film level and cell integrity, we investigated a possible association between the tear cell content and the tear volume. To achieve this goal, we measured the tear collection volume before centrifugation and cell counting, andanalyzed the possible correlation between the two variables. As highlighted in Figure 1, tear volume and cell content were positively associated, with an r value of 0.47 (Figure 1).

To evaluate the performance quality of Countess 3 FL, the precision was measured in terms of both intra-assay and inter-assay variabilities. The intra-assay variability was analyzed by calculating the coefficient of variation (CV) between two different measures of three different samples by the same operator. As summarized in Table 1, the average of individual CVs among repeated measurements performed by the same operator was 4.7%.

For inter-assay variability, CV was calculated among repeated measures on two different samples performed by two different researchers. As reported in Table 2, the average of CVs was 8.7% and 9.7% for Sample 1 and Sample 2, respectively.

### 3.2. HLA-DR and CD3 Quantificationby Countess FL

**Figure 2:**
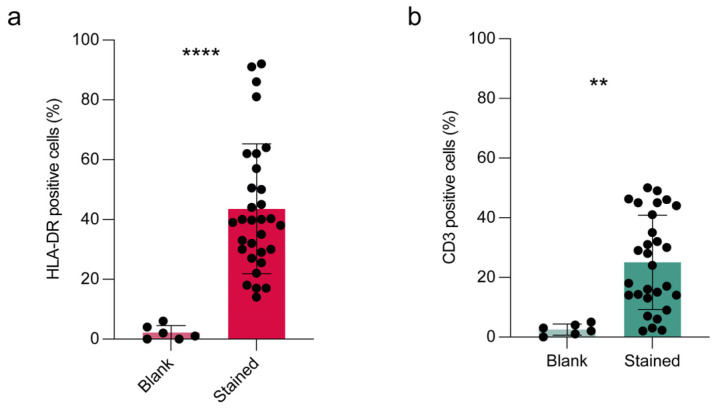
Detection of HLA-DR and CD3 in cells collected from human tear samples by Countess 3 FL. (**a**) HLA-DR and (**b**) CD3 markers were analyzed in all human tear cell pellets collected divided into two groups: blank (sample without antibody incubation, negative control for method specificity) and stained (samples stained with both antibodies by double immunofluorescence). GFP and RFP were selected for fluorescence determination. Results are expressed as percentage of cells positive to antibody staining and reported as mean ± standard deviation of at least two measures of each sample. Statistical analysis was performed by unpaired Student *t*-test; **, *p* <0.01; ****, *p* <0.0001.

Once the Countess 3 FL capacity of counting cells in tear pellets was evaluated, we aimed at investigating whether this benchtop instrument was able to analyze antigen expression by fluorescence detection. The main goal was to evaluate HLA-DR and CD3 expression, for inflammation and lymphocyte monitoring. After establishing the fluorescence staining of both antibodies by direct immunofluorescence (Supplementary Figure S1), we proceeded to Countess 3 FL analysis. Blank samples without fluorescent antibody incubation were included for checking method specificity. HLA-DR expression was detected in all samples and accounted for the 43.6% ± 21 of analyzed samples (Figure 2a), significantly different from blank samples (*p* < 0.0001, unpaired Student *t*-test). These data supported the Countess specificity in HLA-DR determination in tear cell pellets. Similarly, we found a significant difference between blank and stained samples for CD3, which resulted in 25 ± 15% (*p* < 0.01, unpaired Student *t*-test) of analyzed samples (Figure 2b).

**Figure 3:**
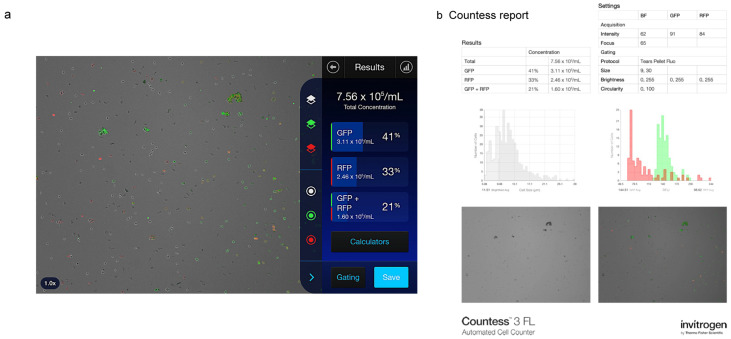
Representative image of an analysis performed with the Countess 3 FL Automated Cell Counter on a sample of human tear cell pellet in both brightfield and fluorescence mode. (**a**) Instrument interface and (**b**) report summarizing all data and relative graphs for cell count, CD3 and HLA-DR marker analysis. Green: green fluorescent protein; red: red fluorescence protein.

A representative image of Countess 3 FL analysis results and relative report is shown in Figure 3.

In order to evaluate the performance quality for fluorescent antibody detection, intra-assay and inter-assay CVs were calculated. Mean CV relative to intra-assay variability of HLA-DR and CD3 were 177 and 2.1, respectively. Data are summarized in Table 3.

Table 4 and Table 5 report CV data relative to inter-assay variability of HLA-DR and CD3 staining, respectively. CV values were all under 10%.

### 3.3. Performance of Countess 3 FL in Comparison to Flow Cytometry

To validate the feasibility of Countess 3 FL and the potential application into a clinical setting, we compared the Countess and flow cytometry in terms of fluorescence quantifications. To this aim, 20 tear samples were analyzed for HLA-DR and CD3 expression with both methods displaying comparable results that corroborated the Countess 3 FL reliability in fluorescent marker detection in tear cell pellets. Further, we found a significant correlation between flow cytometry and Countess 3 FL for HLA-DR detection.

**Figure 4:**
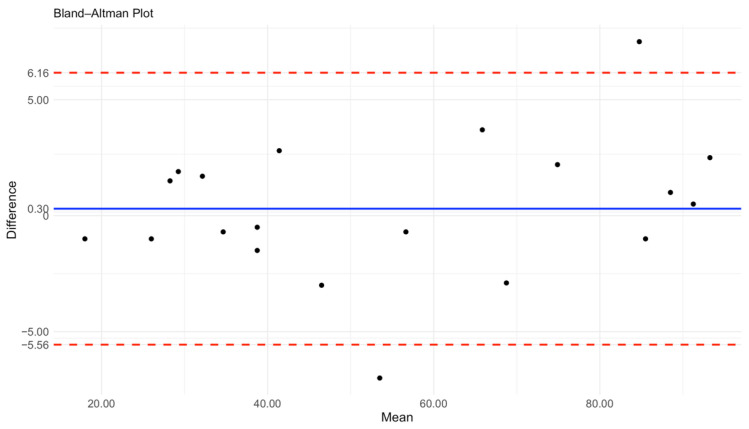
A Bland–Altman plot to detect differences in HLA-DR counts measured using Countess 3 FL and flow cytometry equipment (*N* = 20). The mean difference (blue line) and limit of agreement (red dashed lines) are plotted. The solid line indicates the 95% limits of agreement (mean difference ± 1.96 SD). Black dots indicate the HLA-DR counts results in each sample.

The Bland–Altman plot demonstrates the agreement between the Countess 3 FL automated cell counter and flow cytometry with a R value of 0.99 for cell quantification (Figure 4). The mean difference (bias) between the two methods is 0.3, indicating minimal systematic deviation with Countess measurements being slightly higher than those obtained by flow cytometry.The limits of agreement (LoA) range from −5.56 to 6.16, meaning that 95% of the differences fall within this interval. Given the expected variability in biological samples and measurement techniques, these values are within an acceptable range for clinical application. Overall, the results indicate a strong agreement between the two methods, supporting the use of Countess 3 FL as a reliable alternative to flow cytometry for routine clinical analysis.

**Figure 5:**
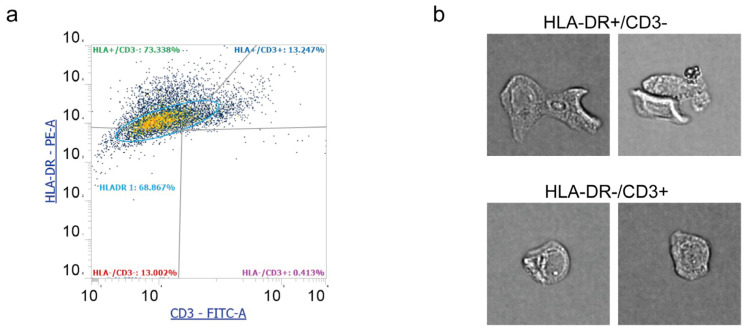
Representative image analysis performed by Attune Cytometric Software on human tear cell pellets. (**a**) Representative plot of flow cytometry assessment of HLA-DR (PE-A) and CD3 (FITC) and (**b**) cell morphology images of both HLA-DR+/CD3− and HLA-DR−/CD3+. Cell size: 15–25 mm in diameter.

To strengthen and enrich data acquired with Countess 3 FL and standard flow cytometry, we performed an additional analysis with Attune Cytometric Software. This facility translates event features into distinct morphometric parameters that can be combined with standard fluorescence and scatter parameters. The percentage of events positive to HLA-DR and CD3 were 39% and 10%, respectively. Figure 5a shows a representative plot of Attune Cytometric analysis. Overall. data were consistent with those obtained from FACSCanto analysis and Countess 3 FL. Further, in Figure 5b it is possible to appreciate cell features and morphology, observing HLA-DR+/CD3− cells with the epithelial shape and elongations. HLA-DR−/CD3+ cells, instead, display rounded morphology, showing the presence of leukocytes in the conjunctiva.

### 3.4. HLA-DR Detection by Countess FL in an In Vitro Model of Hyperosmolar Stress

**Figure 6:**
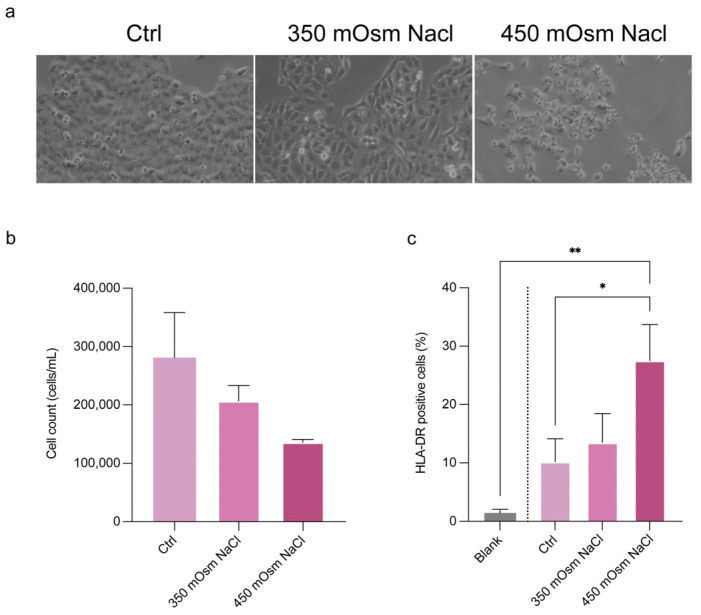
Detection of HLA-DR in a cell model of hyperosmolar stress by Countess FL. (**a**) Cell morphological alterations following exposure to 350 mOsm and 450 mOsm NaCl to mimic the hyperosmolar stress in mild and severe dry eye, respectively. Analysis of (**b**) cell count and (**c**) HLA-DR expression in CCL20.2 exposed to NaCl, measured by Countess. Data are reported as percentages of cells positive to HLA-DR and represented as means of three independent experiments (mean ± sd). Statistical analysis was performed using a one-way Anova test followed by Bonferroni’s test for multiple comparisons. * *p* < 0.05. ** *p* < 0.01. Blank, negative control without antibody incubation; ctrl, control cells without NaCl treatment and stained with antibody; mOsm, milli osmolar; NaCl, sodium chloride; sd, standard deviation.

To strengthen the performance of the automated cell counter method, we tested the HLA-DR detection ability in a conjunctival epithelial cell model. For this goal, CCL20.2 cells were exposed at increasing concentration of NaCl for mimicking hyperosmolar stress as follows: 350 mOsm NaCl for mild dry eye and 450 mOsm NaCl for severe dry eye. In Figure 6a the cell morphological alterations after NaCl stress exposure are shown. After 24 h, we found a slight decrease in cell number (Figure 6b) and a dose-dependent expression of HLA-DR (Figure 6b). A 30% increase of HLA-DR expression was found in CCL20.2 treated with 450 mOsm NaCl (*p* < 0.05 as compared with untreated cells). To check for specificity of detection, the negative control without antibody staining (blank) was included, showing a mean fluorescence of 1.4% (Figure 6c), in accordance with data obtained in patient tear cells.

## 4. Discussion

This study investigated the applicability of the Countess 3 FL for the immunophenotyping and characterization of exfoliated cells obtained from tear samples. The results demonstrate that this instrument provides rapid and reproducible measurements with minimal operator intervention, yielding data comparable to those obtained through conventional flow cytometry—the current standard for similar applications. These findings suggest that the Countess 3 FL may represent a practical tool for routine laboratory analysis, particularly in contexts involving low-volume biological samples such as tears. Our study demonstrated good precision and reproducibility for both total cell counts and immune stained subpopulations (CD3⁺ and HLA-DR⁺), with coefficients of variation consistently below 10%, which are considered very good in terms of statistical significance [14] despite the intrinsic variability and low volume of this biological matrix. Moreover, the concordance with flow cytometry, assessed by the Bland–Altman analysis, confirmed the analytical validity of this tool for quantifying both total and immune labeled cells. These findings support the use of Countess 3 FL not only for research purposes but also as a rapid and clinically applicable tool for monitoring ocular surface inflammation, potentially guiding therapeutic strategies.

Recent advancements in laboratory automation have led to the development of specific analytical instruments for cell quantification in biological fluids, allowing faster and more standardized assessments compared to conventional techniques. As reported by Alcaide Martín et al. [15], several hematological and urine analyzers with dedicated body fluid modules have shown promising performance in low-cellularity fluid, although they remain limited in terms of immunophenotyping capabilities and require trained personnel. Jang et al. [16] evaluated the Microscanner Plus for CD4⁺ T lymphocyte counting and reported repeatability CVs ranging from 2.2% to 6.6%. and inter-operator reproducibility CVs below 5%, confirming its suitability for clinical settings. In this context, the Countess 3 FL represents a novel approach by combining automated total cell counts with dual-color fluorescence-based immunophenotyping, offering a user-friendly alternative to flow cytometry for small-volume biological samples like tears. Similarly, Takahashi [17] demonstrated that the Countess 3 FL was capable of accurately quantifying microalgae, with CVs comparable to hemocytometry but with reduced user bias and faster processing time.

Counting and characterizing cells is a crucial step in many research and clinical applications, from basic cell culture maintenance to more advanced diagnostic analyses. Among the available technologies, automated cell counters and flow cytometers are widely used, each with their own strengths and limitations. Flow cytometry represents the gold standard for advanced cellular analysis, as it can detect multiple surface and intracellular markers, making it ideal for detailed immunophenotyping. However, flow cytometry comes with significant drawbacks. The high cost of equipment and maintenance makes it less accessible for many laboratories. Additionally, the workflow is complex, time-consuming and requires extensive sample preparation, including staining, washing and fluorescence compensation. Moreover, trained personnel are needed to ensure proper setup and data interpretation, as errors in gating or fluorescence overlap can lead to misinterpretation of results. Unlike basic models that only assess cell viability, the Countess 3 FL can analyze fluorescently labeled cells, allowing for 2 to 4color immune-phenotyping. It is cost-effective and accessible, easier to maintain than flow cytometers, making it a great option for consistent and reliable data in labs that don’t need complex and multi-parameter analysis. Finally, it requires less biological material, which is especially useful for limited samples like tear fluid. According to our data on cell pellets obtained from human tears, Countess 3 FL provide accurate and precise results in terms of cell count, displaying a positive correlation with the collection tear volume. Furthermore, Countess 3 FL provides reliable and precise data regarding the expression of HLA-DR and CD3, markers of inflammation and leukocyte, respectively. The robustness of the method was validated by performing parallel analysis on the same group of samples by standard flow cytometry.

To the best of our knowledge, this is the first study to evaluate the feasibility of using an automated cell counter for antigen detection in human tear samples through a fluorescence-based approach. The results are promising and support the potential clinical application of this method, particularly in scenarios where low sample volume poses a major limitation for tear-based diagnostics. Moreover, reducing operator-dependent variability is crucial to improve the accuracy and reproducibility of results. Further technical refinements are therefore warranted to address these limitations and enhance the robustness of the method.

## 5. Conclusions

Data from the present study suggest that the Countess 3 FL may represent an optimal tool for analyzing cells in small tear volumes, particularly within clinical laboratory settings. Its ability to rapidly deliver reliable cell counts and fluorescence-based immunophenotyping enables efficient assessment of ocular surface inflammation, supporting timely and personalized therapeutic decision-making. The Countess 3 FL emerges as a valuable alternative to flow cytometry, especially for routine clinical and research applications where a fast, cost-effective and user-friendly method for analyzing fluorescently labeled cells is required.

Future studies will aim to optimize the protocol further, with a focus on standardizing the detection of additional biomarkers and refining the overall analytical workflow.

## Figures and Tables

**Table 1:** Precision evaluation of cell counting by Countess FL based on intra-assay variability.

Sample	Mean Cell Count	SD	CV (%)	Mean CV (%)
1	5845	170	2.9	
2	6052	295	4.8	4.7
3	2028.75	132.5	6.5	

SD, standard deviation; CV, coefficient of variation.

**Table 2:** Inter-assay variability of cell counting by Countess FL based on operator-dependent reproducibility.

Sample	Researcher	Mean	SD	CV (%)
1	Researcher A	4140	1414.2	8.7
	Researcher B	5710	806	0.7
2	Researcher A	2880	367	9.6
	Researcher B	2615	742	0.58

SD, standard deviation; CV, coefficient of variation.

**Table 3:** Precision evaluation of HLA-DR and CD3 analysis by Countess 3 FL based on intra-assay variability.

Marker	Sample	Mean Positivity (%)	SD	CV (%)	Mean CV (%)
HLA-DR	1	38%	0.005	1.3	1.7
	2	64%	0.005	0.77	
CD3	1	45%	0.005	1.17	2.1
	2	27%	0.005	1.86	

CV. coefficient of variation; SD. standard deviation.

**Table 4:** Inter-assay variability of HLA-DR staining by Countess FL based on operator-dependent reproducibility.

Sample	Researcher	Mean Positivity	SD	CV (%)
1	Researcher A	58%	0.12	2.1
	Researcher B	37%	0.11	0.7
2	Researcher A	53%	0.04	1.8
	Researcher B	54%	0.05	0.58

**Table 5:** Inter-assay variability of CD3 staining by Countess FL based on operator-dependent reproducibility.

Sample	Researcher	Mean Positivity	SD	CV (%)
1	Researcher A	20%	0.07	0.8
	Researcher B	19%	0.08	0.7
2	Researcher A	42%	0.02	7.5
	Researcher B	51%	0.07	0.58

## Data Availability

Raw data will be available upon the reasonable request to the corresponding author.

## Supplementary Materials

The following supporting information can be downloaded at: https://www.mdpi.com/article/10.3390/diagnostics15091124/s1.
