# Supplementary figures and images for: Validation of an Automated Cell Counter Method for HLA-DR and CD3 Expression in Cells Obtained from Low Volume Human Tears
Source: Diagnostics (Basel). 2025 Apr 28;15(9):1124. doi: 10.3390/diagnostics15091124 (PMC12071788; doi:10.3390/diagnostics15091124)

blue: DAPI  
red: HLA-DR  
green: CD3

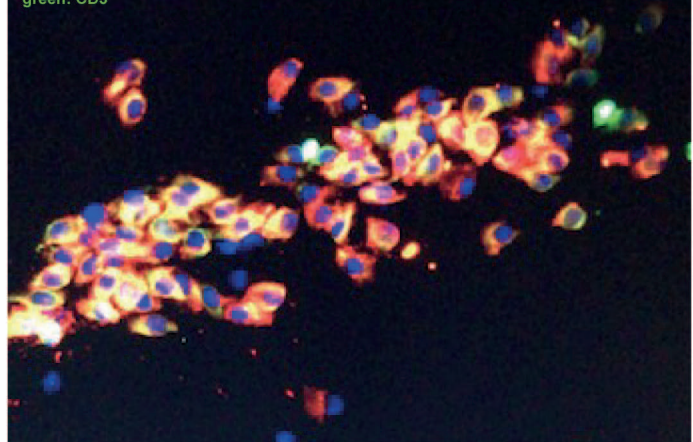

Supplement: Supplementary file 1 [file diagnostics-15-01124-s001.zip › diagnostics-3582547-supplementary.pdf]
